# Effects of Subretinal Gene Transfer at Different Time Points in a Mouse Model of Retinal Degeneration

**DOI:** 10.1371/journal.pone.0156542

**Published:** 2016-05-26

**Authors:** Xufeng Dai, Hua Zhang, Juanjuan Han, Ying He, Yangyang Zhang, Yan Qi, Ji-jing Pang

**Affiliations:** School of Ophthalmology and Optometry, The Eye Hospital, Wenzhou Medical University, Wenzhou, Zhejiang, P.R. China; National Eye Institute, UNITED STATES

## Abstract

Lysophosphatidylcholine acyltransferase 1 (LPCAT1) is necessary for photoreceptors to generate an important lipid component of their membranes. The absence of LPCAT1 results in early and rapid rod and cone degeneration. Retinal degeneration 11 (*rd11*) mice carry a mutation in the *Lpcat1* gene, and are an excellent model of early-onset rapid retinal degeneration (RD). To date, no reports have documented gene therapy administration in the *rd11* mouse model at different ages. In this study, the AAV8 (Y733F)-smCBA-*Lpcat1* vector was subretinally injected at postnatal day (P) 10, 14, 18, or 22. Four months after injection, immunohistochemistry and analysis of retinal morphology showed that treatment at P10 rescued about 82% of the wild-type retinal thickness. However, the diffusion of the vector and the resulting rescue were limited to an area around the injection site that was only 31% of the total retinal area. Injection at P14 resulted in vector diffusion that covered approximately 84% of the retina, and we found that gene therapy was more effective against RD when exposure to light was limited before and after treatment. We observed long-term preservation of electroretinogram (ERG) responses, and preservation of retinal structure, indicating that early treatment followed by limited light exposure can improve gene therapy effectiveness for the eyes of *rd11* mice. Importantly, delayed treatment still partially preserved M-cones, but not S-cones, and M-cones in the *rd11* retina appeared to have a longer window of opportunity for effective preservation with gene therapy. These results provide important information regarding the effects of subretinal gene therapy in the mouse model of LPCAT1-deficiency.

## Introduction

Inherited retinal degeneration (RD) is caused by a group of eye diseases that result from mutations in various genes. There are several modes of inheritance, such as autosomal dominant, autosomal recessive, X-linked recessive, digenic, and mitochondrial. Dominant mutations can arise through loss-of-function, gain-of-function, and dominant-negative mutations, whereas recessive mutations only present a deficiency in functional wild-type gene protein products [1]. Therefore, restorative gene therapy is a tangible approach for treatment of disorders involving recessive loss-of-function mutations [2].

Retinal degeneration 11 (*rd11*) mice have a deficiency in their lysophosphatidylcholine acyltransferase 1 (LPCAT1) enzyme function, due to a recessive mutation in the *Lpcat1* gene [3]. L PCAT1 is a phospholipid biosynthesis and remodeling enzyme that catalyzes the conversion of palmitoyl lysophosphatidylcholine (LPC) to dipalmitoylphosphatidylcholine (DPPC) [4]. DPPC is an important lipid component of the cell membrane [5]. Normally, LPCAT1 expression is reported to rise from postnatal days 2 (P2) to 25 (P25) in mice [6]. The loss of LPCAT1 leads to reduced DPPC levels resulting in membrane disruption, Ca^2+^ influx [7], and eventually cell death. Typical *rd11* mice show early-onset, rapid RD with rod photoreceptors being affected before cones [3]. Mouse mutants with RD provide a valuable resource to discover human disease genes. In North America and China, molecular screening of the *Lpcat1* gene in patients with retinitis pigmentosa or leber congenital amaurosis was performed [3,8], but no disease-causing mutations in the *Lpcat1* gene were identified so far. Though we could not establish disease causality in patients, *Lpcat1* gene remains a candidate for human RD due to the importance of DPPC and lipid metabolism in the retina. It is possible that *Lpcat1* mutations have a pleiotropic effect in human beings. Therefore, further *Lpcat1* mutation screening in patients with RD is still desirable [3].

Gene replacement therapy, especially when performed within the optimum window of time, is currently available to cure monogenic recessive RD, as reported in studies of animal models [9]. The subretinal space is located between the retinal pigment epithelial (RPE) cells and the outer segments of photoreceptor cells. Most of the genes associated with RD are expressed in photoreceptor or RPE cells, both of which are close to the subretinal space, and can be efficiently targeted by subretinal administration of viral vectors [10,11]. In the *rd11* retina, degeneration is caused by mutations in genes expressed in the photoreceptor cells. Using capsid mutant adeno-associated virus (AAV) vector, our previous results of P14-treated *rd11* retinas revealed that only four to five layers of photoreceptors, 74% of the wild-type retinal thickness, and 68% of the wild-type electroretinogram (ERG) responses could be maintained [12]. These results suggested that RD in the mice may have been triggered before P14. To test this hypothesis, in the present study we administered gene therapy at a number of time points, the earliest being P10.

The AAV8 (Y733F) vector was previously reported to express target protein at 10 days post-injection [13]. Prohibiting or delaying RD is very important during these 10 days. Pang et al. previously reported that rearing a *Pde6b* deficient *rd10* mice model in the dark facilitated therapeutic experiments by slowing RD [14,15]. In this study, we tested whether reducing light exposure would improve the outcomes following gene therapy in *rd11* mice. To do this, we raised one group of neonates in dim red light to protect their retinas against light irradiation until 10 days after gene therapy was administered.

In the human retina, cone photoreceptors are responsible for fine resolution, central vision, and color perception. Humans and primate eyes have three types of cone cells that sense either red (long wavelength), green (medium wavelength), or blue (short wavelength) light [16]. There are only two sensitivity maxima in mice, with M-cones having a green-sensitive pigment, and S-cones having an ultraviolet (UV)-sensitive pigment [17,18]. Immunocytochemical evaluation showed these two types of cones have distinctive regional segregation patterns [18]. In *rd11* mice, the retina is characterized by early photoreceptor dysfunction, with rapid rod photoreceptor degeneration followed by cone degeneration [3]. It was shown that early gene therapy could rescue rods and cones [12]; however, no reports to date have documented the residual function or morphology of the *rd11* retina after delayed treatment.

In this study, subretinal gene transfer was performed at four time points to test the best time point for intervention and whether delayed intervention still provides rescue. We tested whether reducing light exposure before and after treatment would improve the rescues in *rd11* mice. Results from this study will provide useful insights into the effectiveness of different subretinal gene therapy approaches for treating RD in the mouse model.

## Materials and Methods

### Animals

Wild-type C57BL/6J mice and the congenic inbred strain of *rd11* mice were acquired from the Jackson Laboratory (Bar Harbor, ME). Approximately 80 *rd11* mice were used in this study. All mice were bred and maintained in the Animal Facilities of Wenzhou Medical University (Wenzhou, China). All mice were maintained on a cycle of 12 hours of light and 12 hours of dark, with an ambient light intensity of 18 lux [18], and had free access to water and food. All experiments were approved by Wenzhou Medical University’s Institutional Animal Care and Use Committee (Permit Number: wydw2014-0072) and were conducted in accordance with the ARVO Statement for the Use of Animals in Ophthalmic and Vision Research.

### Construction of the AAV8 (Y733F)-smCBA-*Lpcat1* Vector

The AAV8 (Y733F) capsid is an AAV serotype 8 capsid with a phenylalanine substituted for the tyrosine at residue 733. AAV8 (Y733F) was used for packaging the vector DNA. AAV8 (Y733F) vector exhibits higher transduction efficiency in photoreceptor cells, and faster onset of expression than other AAV serotypes when injected into the subretinal space [15]. Vector plasmids were constructed by cloning mouse *Lpcat1* cDNA [3] under the control of the small, hybrid CMV-chicken β-actin (smCBA) promoter [14,19] to generate pTR-smCBA-*Lpcat1*. Compared to the full chimeric CMV-chicken β-actin (CBA) promoter, the smCBA promoter has been shown to have identical transduction efficiency and tropism [14]. The capsid mutant AAV vector was packaged and viral titer calculated according to previously reported methods [20].

### Subretinal Vector Injections

The AAV8 (Y733F)-smCBA-*Lpcat1* (10^13^ vector genomes per mL) was used to treat *rd11* mice at P10 (Group A), P14 (Group B, C), P18 (Group D) or P22 (Group E). One microliter of the vector was injected subretinally into one eye of each mouse, and the other eye remained uninjected as a control. Subretinal injections were performed as described previously [10]. We routinely administered a small amount of fluorescein (0.1 mg/mL final concentration) with the vector to visualize the injection and bleb formation. Only animals with no apparent surgical complications were kept for further evaluation, and we retained at least eight mice per group for each experiment. Subretinal injection can lead to retinal detachment between the neuroretina and the RPE layer [10,11]. Immediately following surgery, the retinal location and extent of the subretinal blebs were documented by fundoscopy. Flattening of the subretinal bleb and retinal reattachment occurred within 2–3 days. In groups A, C, D, and E, the mice were maintained in a vivarium with a normal 12-hour light/dark cycling room light (18 lux) [18]. The *rd11* mice of group B were born and reared in a dim red light environment (wavelength > 650 nm, intensity < 5 lux), to avoid exposing their eyes to light until 10 days after gene therapy.

### ERG Recordings

Standard scotopic and photopic ERGs were recorded from anesthetized mice using a Ganzfeld dome fitted with the light emitting diode (LED) stimuli of a ColorDome stimulator (Roland Consult, Wiesbaden, Germany). The scotopic ERG was recorded at 3.0 cd∙s/m^2^ stimulus intensity of white LEDs (450–780 nm). Five responses were recorded and averaged. Following a 10 minute photopic adaptation (30 cd/m^2^ background), green (511 nm) and ultraviolet (UV, 363 nm) LEDs were used as the stimulation light sources for recording the M-cone and S-cone ERGs, respectively [18,21]. As the optimal intensity to mice, the green light was set at 0.75 cd∙s/m^2^, and the UV at 3.00 mW∙s/m^2^ [18]. Recordings were performed in the presence of a constant 30 cd/m^2^ rod-suppressing white background light. Fifty responses to each light source were recorded and averaged. The Ganzfeld stimulus used was a full-field flash with a duration of 2 ms, and the band pass of the amplifiers was 1–500 Hz. The amplitude of the a-wave was measured from the prestimulus baseline to the trough of the ERG while the amplitude of the b-wave was measured from the trough to the peak.

### Retinal Morphometry by Light Microscopic Level

Mice were sacrificed by CO_2_ inhalation after ERG testing. Eyes were enucleated and prepared for morphological analysis. Briefly, eyecups were fixed with 10% formaldehyde in phosphate-buffered saline and embedded in paraffin. Retinas that underwent subretinal injections were sectioned both in rescued (vector coverage) and unrescued (out of vector coverage) regions. Sections (4 μm thick) were stained with hematoxylin and eosin (H&E) before being photographed with a Zeiss bright-field microscope (Axio Imager Z1; Carl Zeiss Meditec, Oberkochen, Germany). Images of the posterior pole retinas were obtained. Corresponding neural retina thickness for each group was compared at an equivalent distance (0.3 mm) from the optic nerve by measuring the distance from the vitreal face of the ganglion cell layer to the apical face of the RPE.

### Immunohistochemistry

Enucleated eyes were fixed overnight in 4% paraformaldehyde at 4°C. After removing the optic nerve head, retinal whole-mounts were obtained by separating the neuroretina from the eyecup. Besides whole mounts, retinas that underwent subretinal injections were sectioned both in rescued (vector coverage) and unrescued (out of vector coverage) regions. Cryosections of 10 μm were examined by immunohistochemistry for protein expression. A primary antibody for LPCAT1 (rabbit anti-mouse LPCAT1, HPA022268; Sigma-Aldrich, St. Louis, MO) was used at a dilution of 1:750. A goat anti-rabbit IgG conjugated to a Cy3 fluorophore (AP187C; Merck Millipore, Darmstadt, Germany) was used as a secondary antibody. The nuclei were stained with 4', 6-diamidino-2- phenylindole dihydrochloride (DAPI) for fluorescence immunohistochemistry. In addition, retinal whole mounts and frozen sections were stained for M-cone opsin and S-cone opsin, or rod rhodopsin, according to our previously described methods [12]. At equal distances (0.3 mm) from the optic nerve, cells labeled for M-cone opsins or S-cone opsins were manually counted to determine the density of the two types of cones in retinal whole mounts [18], each within one field (44,139 μm^2^) at a high magnification (×40). Sections then were mounted with coverslips for imaging by fluorescence microscopy.

### Statistical Analysis

All numerical data is presented as the mean ± the standard deviation (SD). Statistical analyzes were performed with SPSS statistical software (ver. 18.0, IBM Corporation, Armonk, NY). One-way ANOVA with a least significant difference (LSD) post hoc test was used for comparison among groups for measurement data. Differences were defined as significant at *P* < 0.05.

## Results

### Postnatal Retinal Development and Degeneration in *rd11* Mouse

**Fig 1:**
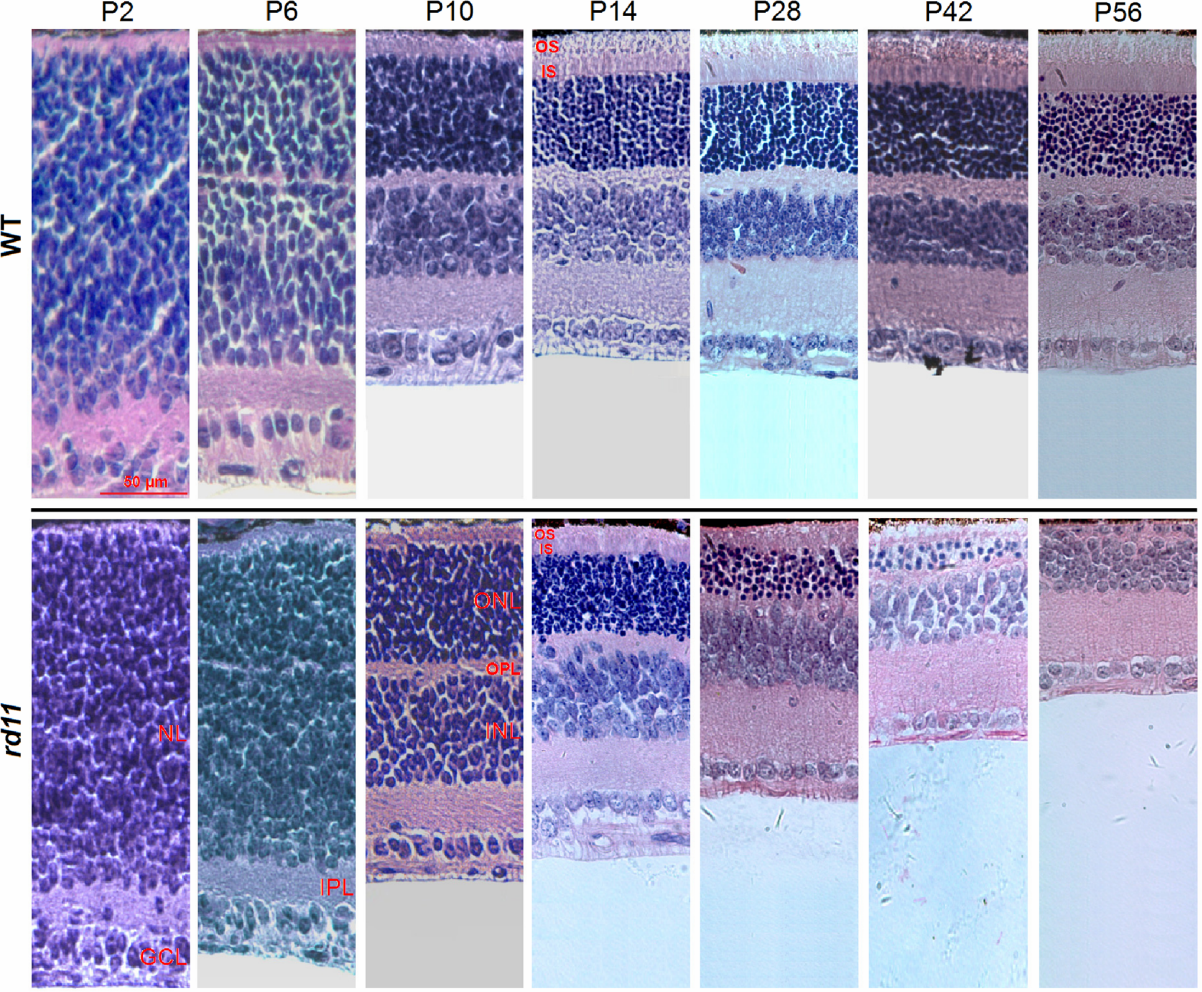
Light microscopy of developing mouse retina after birth. The *Lpcat1* deficient *rd11* (lower row) and WT C57BL/6J retinas (upper row) had similar shape and thickness from P2 to P14. The ONL was completely differentiated from the NL at approximately P10. In WT retinas, the OS reached mature length with loose discs attached to the RPE at approximately P14. After P14, the thickness of the *rd11* retina was markedly reduced due to RD. Age-matched WT C57BL/6J mice were used as controls. NL, neuroblastic layer; GCL, ganglion cell layer; IPL, inner plexiform layer; ONL, outer nuclear layer; OPL, outer plexiform layer; INL, inner nuclear layer; IS, inner segments; OS, outer segments; P, postnatal day; WT, wild-type.

We first characterized the course of photoreceptor maturation and degeneration in postnatal mice at the histological level. Fig 1 shows the retinal segments of the posterior pole. Retinal development occurred postnatally with gradual maturation of photoreceptors. At P2, both strains of mice (*rd11* and control) appeared to have similar retinal structure across sections, which consisted of only a neuroblastic layer (NL), an outer layer, and an inner ganglion cell layer (GCL). Differentiation of the NL showed the outer nuclear layer (ONL) and the inner nuclear layer (INL) started to be gradually divided by an outer plexiform layer (OPL) around P6. Photoreceptors in the outer retina were completely differentiated from the NL by about P10. At P14, the outer segments (OS) of photoreceptors displayed mature length with loose discs attached to the RPE (Fig 1). The loose connection between the OS and the RPE was easily broken after subretinal injection [10,11]. After P14, the thickness of *rd11* retinas was markedly reduced with progressive thinning and loss of the OS and the ONL due to RD (Fig 1).

### Retinal Area Covered by Subretinal Injections at Different Time Points

**Fig 2:**
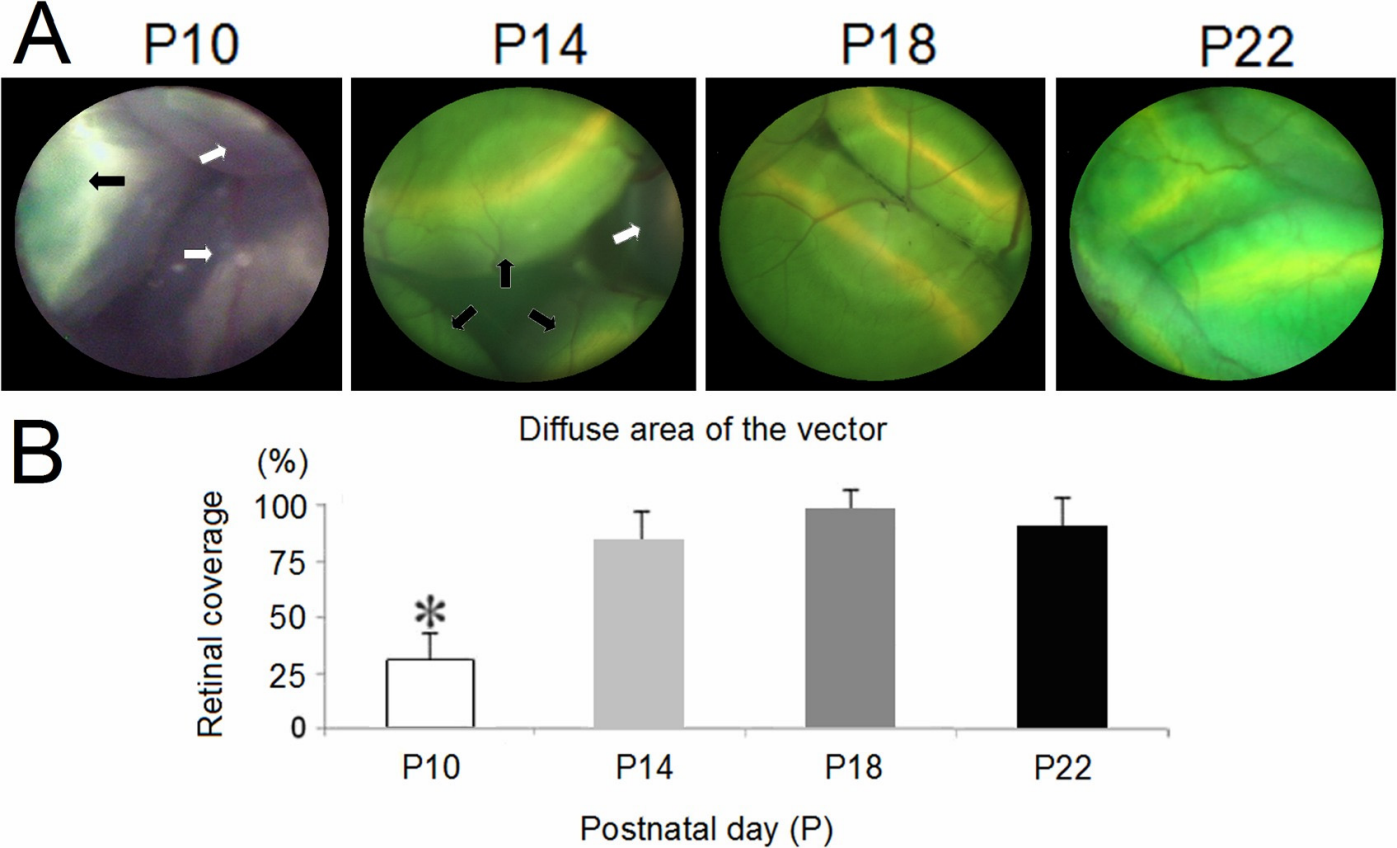
The extent of the diffusion of subretinally injected vector in *rd11* mouse retinas. In the treated *rd11* eyes, 1 μL of the AAV8 (Y733F) vector was injected subretinally at postnatal day 10 (P10), 14, 18, or 22. A small amount of green fluorescein was added to help visualize the diffusion of the vector (A, black arrows). White arrows showed the area there was no accumulation of vector. *Statistical analysis (B) indicated a significant difference in the percentage of the retina covered by the vector after diffusion (*P* < 0.001) in the P10 group, compared to the P14, P18, and P22 groups. No statistical difference in the percentage of the retina covered by vector was found between the P14, P18, and P22 treated mice (*P* > 0.05). Columns and bars represent mean ± SD (n = 8 mice).

Subretinal injection can lead to retinal detachment between the neuroretina and the RPE layer [10,11]. To differentiate between blebs filled versus not filled with injected vector, 0.1% sodium fluorescein (green) was added to the vector solution. The extent of diffusion was measured by counting those blebs with green color underneath [10]. In the treated *rd11* eyes, retinal blood vessels could clearly be seen on the blebs with green dye underneath (Fig 2A), suggesting that the 1 μL of injected vector was in the subretinal space [22]. The subretinal vector in the P10 group was localized around the injection area and diffused to cover only 31.3% ± 11.6% of the whole retina, an area much smaller than that covered by the vector in other groups (n = 8 mice, *P* < 0.001; Fig 2B). There was no significant difference (*P* > 0.05) in the area of the retina covered by diffusing vector between the P14 group (84.4% ± 12.9%), P18 group (96.9% ± 8.8%), and P22 group (90.6% ± 12.9%).

### Rescue of Retinal Morphology after AAV8 (Y733F) Vector Treatments on Different Postnatal Days

**Fig 3:**
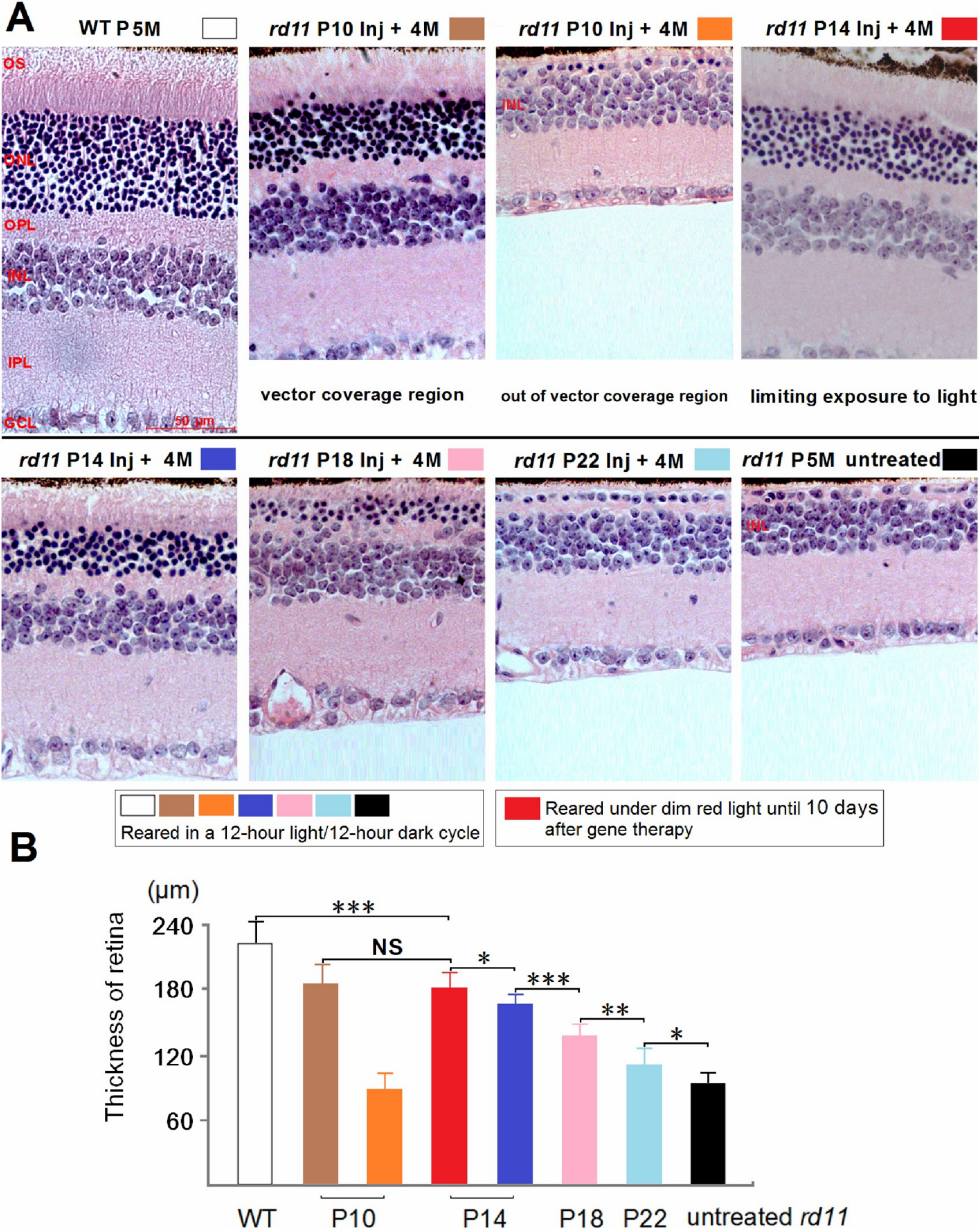
Retinal sections of *rd11* mice treated with gene therapy at different postnatal days. (A) Representative images of H&E-stained retinal sections at 4 months after treatments in P10 to P22-treated groups. Due to the limited diffusion of the vector, whole retinas of the P10 group had both rescued (vector coverage) and unrescued (out of vector coverage) areas. (B) Averaged retinal thicknesses in age-matched WT C57BL/6J, P10 to P22-treated, and untreated *rd11* eyes (n = 8 mice). Mice were born and reared under normal, cyclic light conditions (12 hours light, 12 hours dark), with the exception of one group of P14 treated *rd11* neonates that was reared under dim red light to protect their retinas from light until 10 days after gene delivery. At an equivalent distance (0.3 mm) from the optic nerve, retinal thickness was measured from the vitreal face of the GCL to the apical face of the RPE. Age-matched C57BL/6J and untreated *rd11* mice were as controls. P, postnatal day; Inj, injected; M, months. *indicates *P* < 0.05, **indicates *P* < 0.01, ***indicates *P* < 0.001, NS = no statistical difference.

At 4 months after treatment, the *rd11* mice were sacrificed and eyes enucleated for histology. With H&E staining, we examined the retinal sections of *rd11* mice treated at P10, 14, 18, or 22 (Fig 3A). The rescued area of each retina was correlated with the subretinal coverage of the vector. In P10 treated eyes, the rescued area was localized around the injection area, with no more than one third of the whole retina being affected (Figs 2 and 3A). A significant difference was found in retinal thickness between the area of vector coverage (183 ± 17 μm) and the area outside of vector coverage (88 ± 15 μm) in the P10-treated eyes (n = 8 mice, *P* < 0.001; Fig 3B). In the rescued area, five to six layers of photoreceptors remained. Among the P18 to P22 treatment groups, the rescued thickness of the ONL, inner segment (IS), and the OS was markedly reduced. There were statistically significant differences in retinal thickness between the normal-light treated P14 group (165 ± 9 μm), the P18 group (135 ± 12 μm), and the P22 group (109 ± 15 μm, n = 8 mice, *P* < 0.01 or *P* < 0.001). In both the P14 groups, subretinal vectors diffused to cover 84% of the retina (Fig 2B), but the rescued retinal thickness was greater in the group that had limited exposure to light (180 ± 14 μm, n = 8 mice, *P* < 0.05), as shown in Fig 3B. Limiting exposure to light increased the rescued retinal thickness to approximately 82% of the retinal thickness of wild-type (WT) C57BL/6J mice (220 ± 23 μm). No statistical difference in rescued retinal thickness was found between the light-limited P14 group and the P10 group (n = 8 mice, *P* > 0.05).

### LPCAT1, rhodopsin, and Cone-opsin Expression Following AAV8 (Y733F) Vector Treatments on Different Postnatal Days

**Fig 4:**
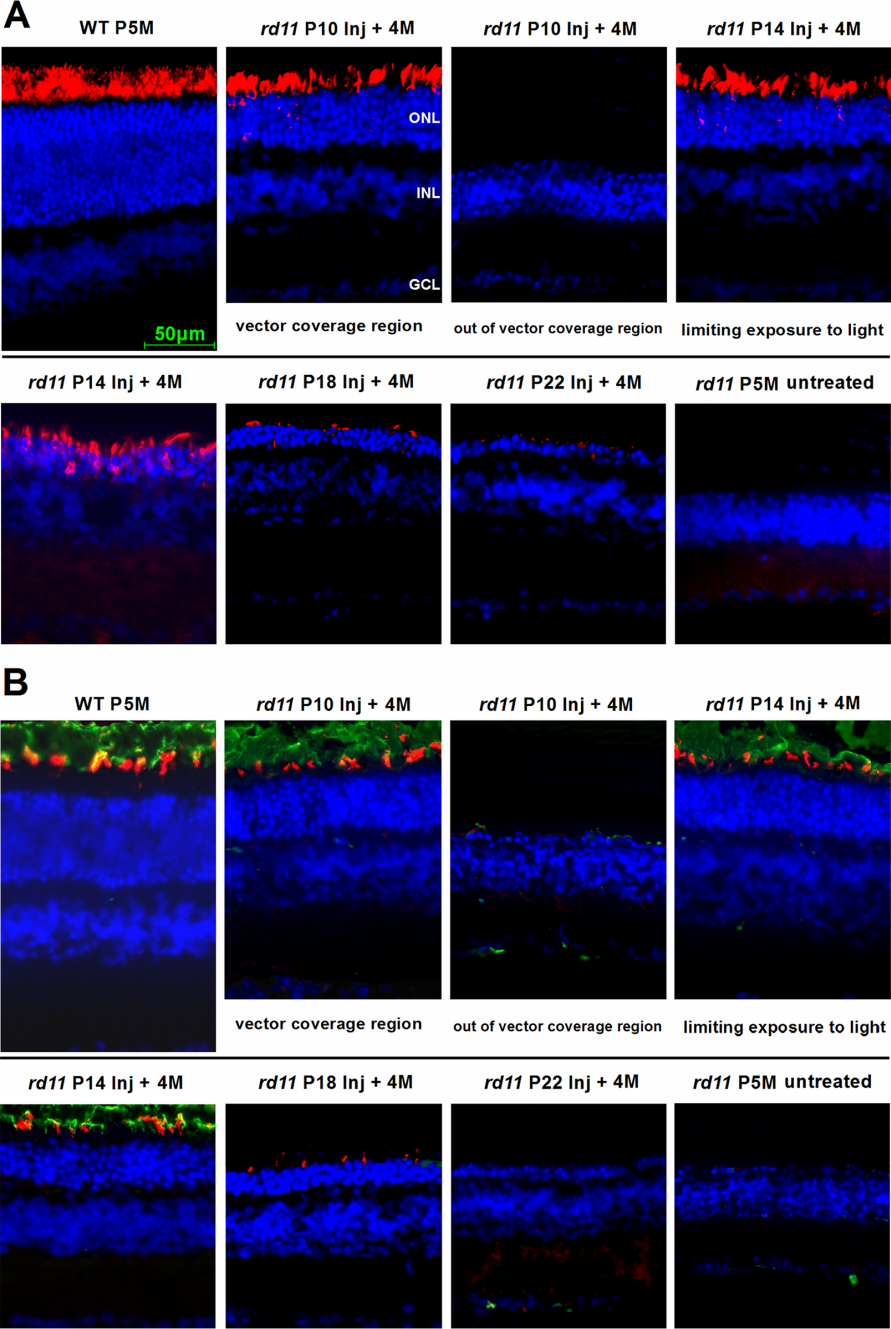
LPCAT1, rod rhodopsin, and cone opsin expression following AAV8 (Y733F) vector treatments on different postnatal days. Retinal images of the posterior pole segments were collected at a distance of 0.3 mm from the optic nerve. (A) LPCAT1 immunostaining (red) of *rd11* retinas at 4 months after treatment in P10 to P22-treated mice. (B) Double staining of rod rhodopsin (green) and cone opsin (red) of *rd11* retinas at 4 months after treatments. Due to limited diffusion of the vector, the retinas of the P10 treated group had both rescued (vector coverage) and unrescued (out of vector coverage) areas. Age-matched wild-type C57BL/6J and untreated *rd11* mice were used as controls. Nuclei were stained with DAPI (blue). P, postnatal day; Inj, injected; M, months.

At 4 months after treatment, LPCAT1 expression was assayed by immunohistochemistry. We examined the retinal cryosections of *rd11* mice treated at P10, 14, 18, or 22. In the P10 group, the photoreceptor-specific gene expression was localized around the injection area with no more than one third of the whole retina being affected. The regional LPCAT1 expression of each retina was correlated with the subretinal area covered by the injected vector, and was limited to photoreceptors (Figs 2 and 4A). Subretinal vectors in the two P14 groups diffused to cover approximately 84% of the whole retina (Fig 2), and the LPCAT1 expression was increased in the P14 group that had limited exposure to light (Fig 4A). Among P18 to P22 treatments, the rescued LPCAT1 expression was markedly reduced. No LPCAT1 expression was observed in the paired untreated retinas from the same *rd11* mice (Fig 4A). Fig 4 shows the retinal area of the posterior pole, which was at a distance of 0.3 mm from the optic nerve. Similar results were found for rod rhodopsin and cone opsin, which were located in the OS. Their expression was detected in the early treatment groups, and was enhanced to levels similar to those of normal C57BL/6J retina by limiting light exposure. However, in the P22 treated *rd11* retinas, expression of rod rhodopsin and cone opsin were not detected (Fig 4B).

### ERG Responses Rescue after AAV8 (Y733F) Vector Treatments on P10 to P22

**Fig 5:**
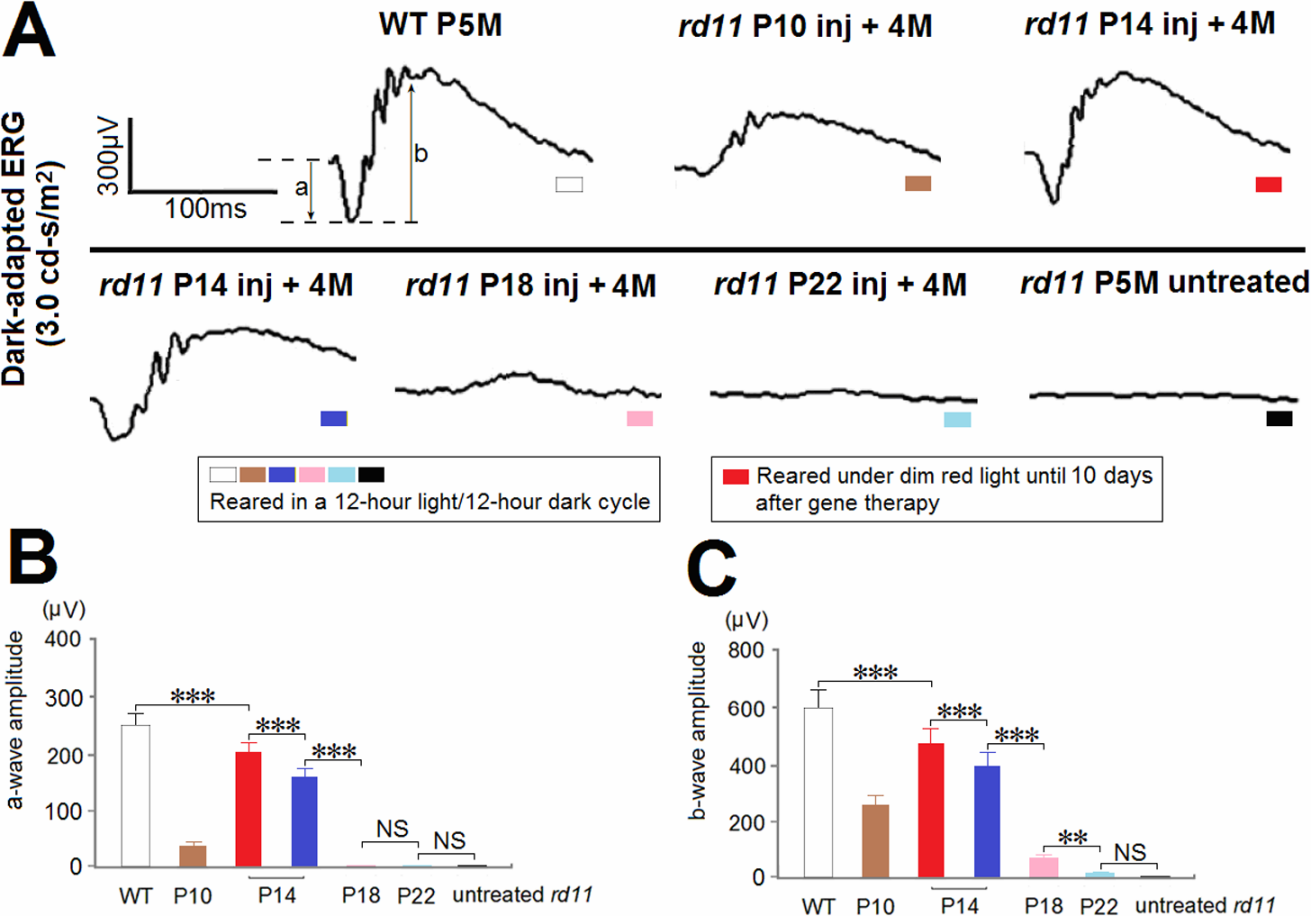
Dark-adapted ERGs from *rd11* mice following AAV8 (Y733F) vector treatments on different postnatal days. (A) Representative scotopic ERGs elicited at 3.0 cd∙s/m^2^ flash intensity in *rd11* eyes 4 months after treatments at P10, 14, 18, or 22. Scotopic a-wave (B) and b-wave amplitudes (C) elicited at 3.0 cd∙s/m^2^ intensity were compared among age-matched wild-type C57BL/6J, P10 to P22 treated, and untreated *rd11* eyes (n = 8 mice). Age-matched wild-type C57BL/6J and untreated *rd11* mice were used as controls. P, postnatal day; Inj, injected; M, months. **indicates *P* < 0.01, ***indicates *P* < 0.001, NS = no statistically significant difference.

Before eyes were enucleated for histology, full-field ERGs were recorded as the response of the whole retina (Fig 5A). Under dark adaptation, the bright stimulus was set at a 3.0 cd∙s/m^2^ intensity to excite both rods and cones [23]. Due to limited diffusion of the vector and partial rescue of the morphology, the *rd11* mice in group P10 showed smaller amplitudes in their a-waves and b-waves compared to those of the normal-light reared P14 group (Fig 5B and 5C and Table 1). Among P14 to P22 treatments, the ERG amplitudes of the normal-light reared P14 group mice were the highest (lower than that of wild-type and light-limited retinas) followed by group P18, and group P22. No a-wave responses were detected in the P18 and P22 treated eyes (Fig 5A and 5B and Table 1). In the P14 group, limiting exposure to light increased scotopic ERG responses to levels approximately 80% of wild-type retinas (Fig 5B and 5C and Table 1).

**Table 1:** Dark-adapted ERG (3.0 cd∙s/m^2^) Amplitudes from *rd11* Mice at 4 Months after Treatment.

		*rd11* mice						P-value	
Amplitude, μV	WT	P10 (A)	P14 (B)	P14 (C)	P18 (D)	P22 (E)	Untreated	P10 (A) vs. P14 (C)	P14 (B) vs. P14 (C)
**A-wave**	**250 ± 21**	**38 ± 6**	**203 ± 17**	**158 ± 16**	**0 ± 0**	**0 ± 0**	**0 ± 0**	**<0.001**	**<0.001**
**B-wave**	**590 ± 61**	**249 ± 35**	**470 ± 50**	**390 ± 44**	**75 ± 9**	**16 ± 3**	**0 ± 0**	**<0.001**	**<0.001**

The AAV8 (Y733F) vector was used to treat *rd11* mice at P10 (Group A), P14 (Group B, C), P18 (Group D) or P22 (Group E). Groups A, C, D, and E were with normal light exposure, but group B with limited light exposure.

Data presented as mean ± standard deviation. n = 8 mice for each group examined. P values calculated using a multiple comparison procedure (LSD test).

### Difference between M-cone and S-cone opsins after AAV8 (Y733F) Vector Treatments on P10 to P22

**Fig 6:**
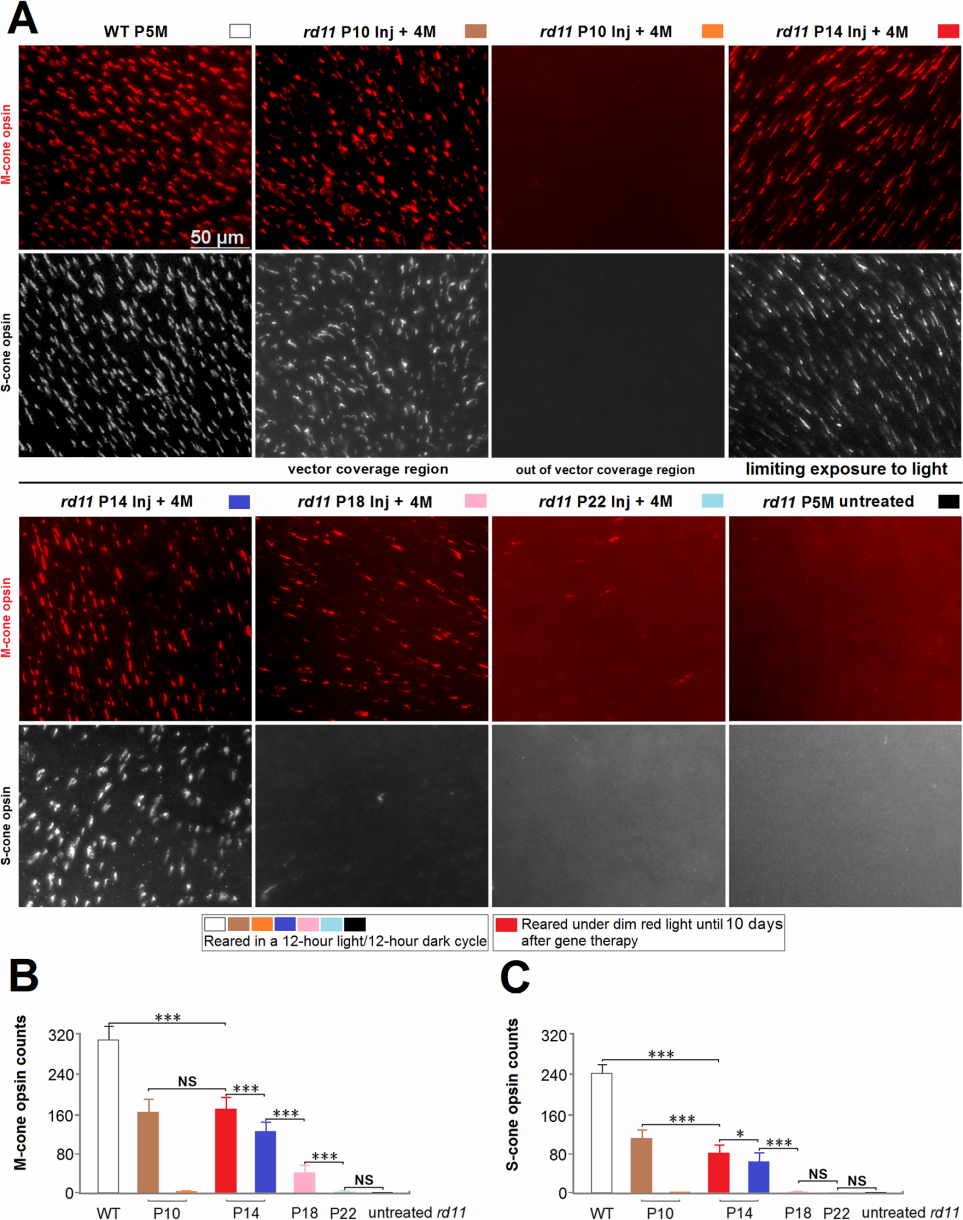
M-cone and S-cone opsins preservation in retinal whole mounts after AAV8 (Y733F) vector treatments at different postnatal days. Retinal M-cone opsins (red) and S-cone opsins (white) of the posterior pole segments were imaged (A) at a distance of 0.3 mm from the optic nerve, and counted (B, C) within one field (44,139 μm^2^) at a high magnification (×40). Due to limited diffusion of the vector, the retinas of the P10 group mice had both rescued (under vector coverage) and unrescued (out of vector coverage) areas. Age-matched wild-type C57BL/6J and untreated *rd11* mice were used as controls. P, postnatal day; Inj, injected; M, months. *indicates *P* < 0.05, ***indicates *P* < 0.001, NS = no statistical difference.

At 4 months after treatment, expression of M-cone and S-cone opsins were assayed by immunostaining (Fig 6A). We examined the retinal whole mounts of *rd11* mice treated at P10, 14, 18, or 22. In the P10 group, photoreceptor-specific gene expression was localized around the injection area with no more than one third of the whole retina being affected. The regional expression of M-cone and S-cone opsins within each retina was in accordance with the subretinal coverage of vector (Figs 2 and 6A). Subretinal vectors in the two P14 groups covered approximately 84% of the whole retinal area (Fig 2B) and limiting exposure to light increased the cone opsins density (Fig 6). Cone degeneration progressed very quickly among P18 to P22-treated groups, spreading from the peripheral to central quadrants in the *rd11* retinas. Fig 6A shows the central retina of the posterior pole, which was 0.3 mm from the optic nerve. Cone-opsin densities from P14 to P22 treated mice (Fig 6A) were decreased. In P18 treated *rd11* eyes, about 15% of wild-type M-cone opsins (Fig 6A and 6B), but no S-cone opsins (Fig 6A and 6C), were observed. Almost no M-cone or S-cone opsins expression was detected in the P22 treated *rd11* retinas, or untreated *rd11* retinas.

### M-cone and S-cone ERGs Rescue after AAV8 (Y733F) Vector Treatments on Different Postnatal Days

**Fig 7:**
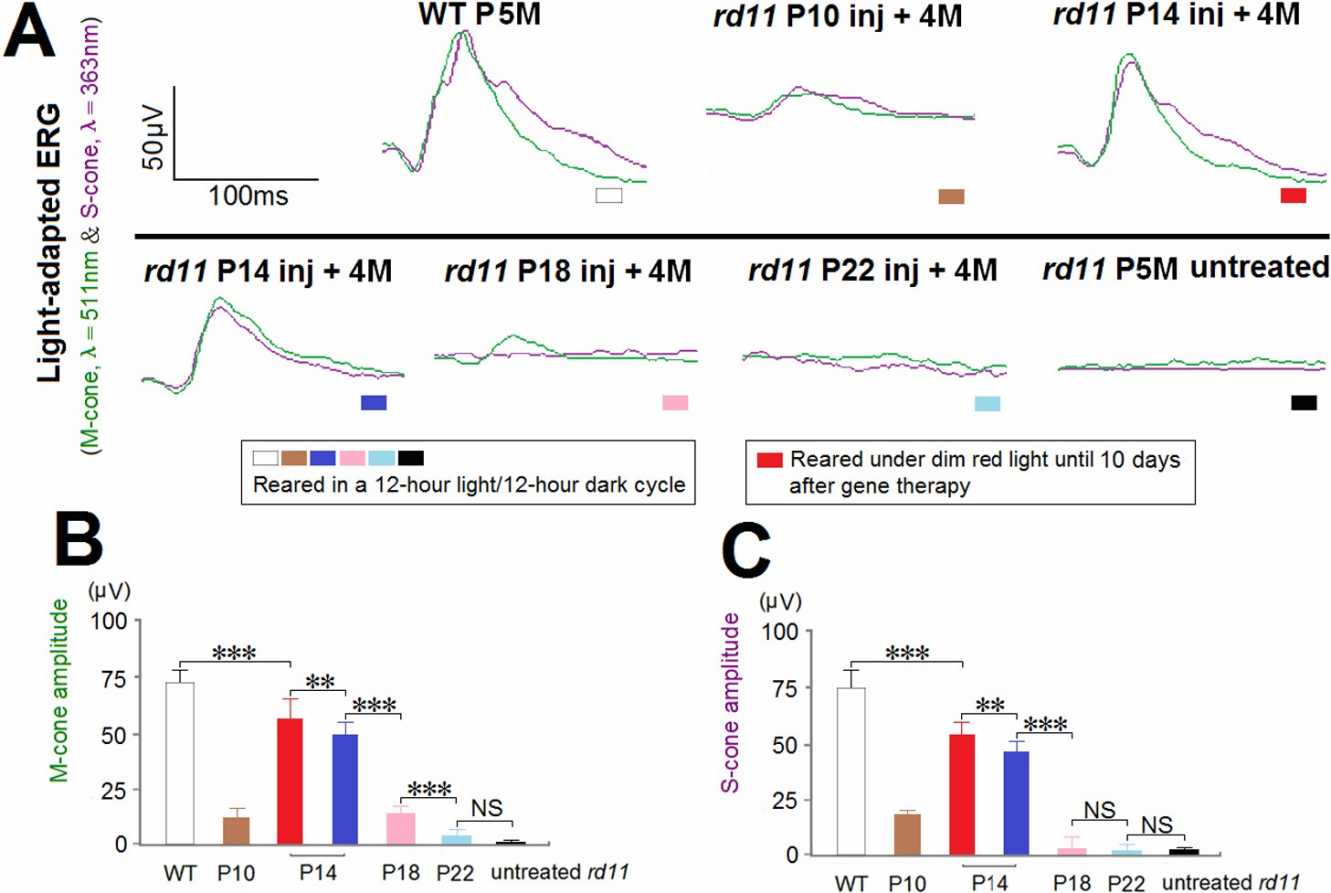
M-cone and S-cone ERGs from *rd11* mice following AAV8 (Y733F) vector treatments on different postnatal days. (A) Photopic ERGs in *rd11* eyes, 4 months after treatments at P10, 14, 18, or 22. M-cone ERGs were recorded at a green light intensity of 0.75 cd∙s/m^2^ while S-cone ERGs were recorded at a UV light intensity of 3.00 mW∙s/m^2^. M-cone (B) and S-cone ERG amplitudes (C) were compared between age-matched wild-type C57BL/6J, P10 to P22 treated, and untreated *rd11* eyes (n = 8 mice). Age-matched wild-type C57BL/6J and untreated *rd11* mice were used as controls. P, postnatal day; Inj, injected; M, months. **indicates *P* < 0.01, ***indicates *P* < 0.001, NS = no statistical difference.

To confirm that preservation of cone opsins correlated with improvement of function, green light (λ = 511 nm) and UV light (λ = 363 nm) stimuli were used to distinguish between activities of the two cone subtypes [18]. As the optimal intensity for mice, the green light was set at 0.75 cd∙s/m^2^, and the UV at 3.00 mW∙s/m^2^ [18]. Full-field ERGs can only represent responses of the whole retina. Therefore, due to the limited diffusion of the vector and partial rescue of the morphology, P10 treated *rd11* mice showed smaller amplitudes in M-cone (12 ± 4 μV) and S-cone (18 ± 2 μV) ERGs compared to normal-light exposed P14 treated *rd11* mice (M-cone ERG: 49 ± 6 μV, S-cone ERG: 46 ± 5 μV; n = 8 mice, *P* < 0.001; Fig 7). Among P14 to P22 treatments, M-cone and S-cone ERG amplitudes were maximal for the light-exposed P14 group, but these were still lower than those of the ERG amplitudes in wild-type and light-limited P14 group mice. The values for the light-exposed P14 group were followed by those for the P18 group (M-cone ERG: 14 ± 3 μV, S-cone ERG: 3 ± 5 μV), and the P22 group (M-cone ERG: 4 ± 3 μV, S-cone ERG: 2 ± 3 μV). No M-cone or S-cone ERGs could be detected in untreated *rd11* mice (Fig 7). Between the two P14 groups, M-cone and S-cone ERG amplitudes were elevated to 56 ± 9 and 53 ± 6 μV, respectively, in the group that had limited exposure to light (n = 8 mice, *P* < 0.01; Fig 7B and 7C). Limiting exposure to light increased photopic ERG responses to levels approximately 75% of those from wild-type retinas (M-cone ERG: 72 ± 6 μV, S-cone ERG: 74 ± 7 μV).

## Discussion

There are many cell types in the neonatal mice retina, and it has been shown that cell generation is in a temporally regulated manner [24,25]. Located in the outer retina, photoreceptors were found to be completely differentiated from NL at about P10 (Fig 1). In this study, an AAV8 (Y733F) containing minimal chicken *β*-actin promoter/CMV enhancer (smCBA) was used to deliver and express *Lpcat1* in *rd11* retinas. Compared to the CBA, the smCBA promoter has been shown to have identical transduction efficiency and tropism (S1 Fig). With a ubiquitous promoter, the vector was delivered at P10, when the well-differentiated photoreceptors could be specifically targeted. Compared with an earlier P2 subretinal injection [26], P10 delivery was more therapeutically effective because injection-related damages that can result from P2 treatment, such as severe cornea-iris adhesion, corneal opacity, traumatic cataracts, or retinal hemorrhage were reduced by the later treatment. It was reported that early injection through intravitreal route [27,28] could provide effective rescue with less retinal trauma, and systemic delivery of AAV in neonatal mice resulted in earlier onset of proteins expression [28]. Intravitreal administration route can be a clinically relevant method of gene delivery to the outer retina depending on the vector used and the disease [29,30].

Compared to gene delivery at P14, P10 treatment with the same vector increased regional retina thickness from 75% to 82% of the wild-type levels, and 5–6 layers of photoreceptors remained in the rescued area (Fig 3). In addition to structural preservation, expression of photoreceptor-specific proteins such as LPCAT1, rod rhodopsin, and cone opsin was preserved within the rescued area to levels similar to those in normal C57BL/6J retinas. However, the disadvantage of P10 treatment was the limited area accessed by the subretinally injected vector, because of the difficulty in detaching a significant fraction of the mouse retina. The limited regional rescue following P10 treatment resulted in a smaller overall rescue of scotopic ERGs compared to the P14 group, in which 1 μL of subretinal vector detached about 84% of the retina (Fig 2). Therefore, subretinal gene administration at P10 was a useful method for regional, but not whole retinal treatment.

The fluorescein-containing fluid was injected into the subretinal space to induce retinal blebs [31,32]. Using microscopy, the extent of subretinal vector diffusion was measured based on the presence of green blebs. In this study, the postnatal development and maturation of OS of photoreceptors seemed to be associated with coverage by the subretinally injected vector. At approximately P10, photoreceptors showed a short and immature OS with dense discs attached to the RPE. Following injection of 1 μL of vector solution, the detachment area of the retina was confined to 31% of the whole retina. Photoreceptors showed a mature OS with loose discs at P14 (Fig 1). At this time, the loose conjunction between OS and RPE was easily detached after subretinal injection, and 1 μL of the injected vector could diffuse to cover about 84% of the whole retina. Previously, we found that one injection using 2 to 3 μL of vector solution did not increase retinal coverage, but caused one big bleb, elevated intraocular pressure, and more injection-related irreversible damage [10]. After P14, the thickness of *rd11* retinas was markedly reduced due to RD, but the degeneration did not affect the area of diffusion of the vector solution.

Early treatment and good vector-diffusion is important to treat the mice model of rapid-progression RD. In human patients, however, the rate of photoreceptor loss may be relatively slower; therefore the window of opportunity for treatment is likely extended. Although it is difficult to extrapolate directly the variable timing of disease onset and progression in a single mouse model to human patients, it is reasonable that the application of gene therapy to rescue the defect should be done as early as would be feasible in the clinical setting.

Gene therapy has been marginally effective, or ineffective, in cases where RD starts too early or progresses too rapidly [33–35]. AAV vectors of different serotypes have been developed to help resolve this problem. These include the AAV8 and AAV5 with the capsid surface-exposed tyrosine residues mutated to phenylalanine (Y-F) that minimize the interval between transduction and transgene expression [13,15]. Sun et al. reported that murine models showing rapid RD were refractory to treatment with AAV5, but responsive to treatment with the AAV8 serotype [36]. In the *rd10* mouse model of RD caused by *Pde6b* mutation, long-term vision was successfully preserved using the capsid mutant AAV8 vector [15]. The AAV8 vector was previously reported to express target protein at 10 days post-injection [13]. In *Lpcat1* deficient *rd11* mice, when RD was delayed by limiting light exposure during the 10 days post-injection, retinas recovered more structurally and functionally following gene therapy. In the light-limited P14 group, retinal thickness was rescued to the same extent as in the P10 group. The biochemical and structural preservation of retinas in light-limited P14 *rd11* mice also led to better electrophysiological function in the rod and cone systems. Although the mechanism involved is not completely understood, dark-rearing is a reliable way to delay RD in the critical 10 days before recovering expression of *Lpcat1*.

In this study, reducing light exposure could improve the outcomes following gene therapy in *rd11* mice. We chose only one time-point to perform ERG test to prevent or decrease as much as possible bright-light induced degeneration. With regard to mice, 4-month after treatment can be considered a relatively long-term followup time-point. After ERG testing (bright-light exposure), the treated mice were sacrificed as soon as possible to perform other analyses.

As the most commonly used model to study blindness, mice have retinas containing less than 3% cones [37]. Earlier electrophysiological and immunocytochemical evaluations showed there were two classes of cones in mouse retinas, having peak wavelength sensitivities of about 511 nm (M-cones) and 359 nm (S-cones) [17,38]. The S-cone pigment of mice is, in fact, a UV pigment [21,39]. Therefore, in mice it seems that lens absorption is not a barrier to UV-based vision [17]. Because cones are primarily responsible for daytime and color vision in humans, rescuing cone photoreceptor function is an essential component of any successful treatment for RD. Unlike the human retina, in which the density of cones is very high in the central macula (fovea), the mouse retina has a relatively even distribution of M-cones and S-cones, although there are more M-cones in the superior retina and more S-cones in the inferior retina [18]. In the P14 to P22 treatments, the rapid loss of M-cone and S-cone opsins expression in *rd11* mice suggests earlier treatment may yield greater preservation of cone function. Relative to M-cone ERGs, early treatment resulted in slightly higher S-cone responses (Fig 7). However, it was difficult to detach a significant fraction of the mouse retina prior to P14 via a subretinal injection. Similar to the rods of neonatal *rd11* retinas, the cones recovered better when exposure to light was limited. Furthermore, P18-treated *rd11* retinas rescued approximately 15% of wild-type M-cone opsins, but not S-cone opsins (Fig 6), and accordingly only M-cones produced ERG responses in these retinas. The potential value of partial preservation of M-cone function following gene therapy at this later stage of RD, especially in the central retina, should not be underestimated. Our results provide another evidence for the rescue of cone function by subretinal administration of gene therapy [18,40–44].

LPCAT1 promotes the conversion of LPC to DPPC [4], and the latter is an important lipid component of the cell membrane that plays numerous biological roles in endocytosis, signaling, and neuroprotection [45,46]. Retinal photoreceptors contain a modified primary cilia structure (referred to as the OS) with highly specialized membrane discs that harbor opsin molecules and other proteins required for phototransduction. Following gene therapy, high level of LPCAT1 expression was localized to photoreceptors, which may be due to the abundant membrane discs in the OS. Different from photoreceptors, here LPCAT1 expression in RPE cells may be lower than the level that can be detected by immunofluorescence staining. The LPCAT1 protein is essential for the survival of photoreceptor cells in the retina [3]. The *rd11* mouse strain carries an *Lpcat1* mutation, and shows degeneration of only the photoreceptor cells, whereas other retinal neurons have subtle changes [3]. Although the importance of LPCAT1 in the retina is established, the biochemical mechanism of photoreceptor cell death caused by the loss of LPCAT1 remains unknown. As a murine model of rod-cone degenerations, further investigations are also required to precisely delineate whether the cone cell death in *rd 11* mice is secondary to rod death or it is the direct effect of the gene mutation.

In summary, we have shown that the outcomes of subretinal gene therapy are age and disease-stage dependent. Early treatment with limited exposure to light improved the outcome for *rd11* eyes, which suffer early-onset, rapid RD. Compared to S-cones, M-cones in the mice appeared to have a longer window for gene therapy intervention. These results provide important information regarding the effects of subretinal gene therapy in the mouse model of LPCAT1-deficiency.

## Supporting Information

S1 Fig AAV8 (Y733F)-mediated GFP expression in retinal frozen-section following subretinal injection of AAV8 (Y733F)-smCBA-GFP. (TIF)
