# Supplementary figures and images for: Effects of Subretinal Gene Transfer at Different Time Points in a Mouse Model of Retinal Degeneration
Source: PLoS One. 2016 May 26;11(5):e0156542. doi: 10.1371/journal.pone.0156542 (PMC4882044; doi:10.1371/journal.pone.0156542)

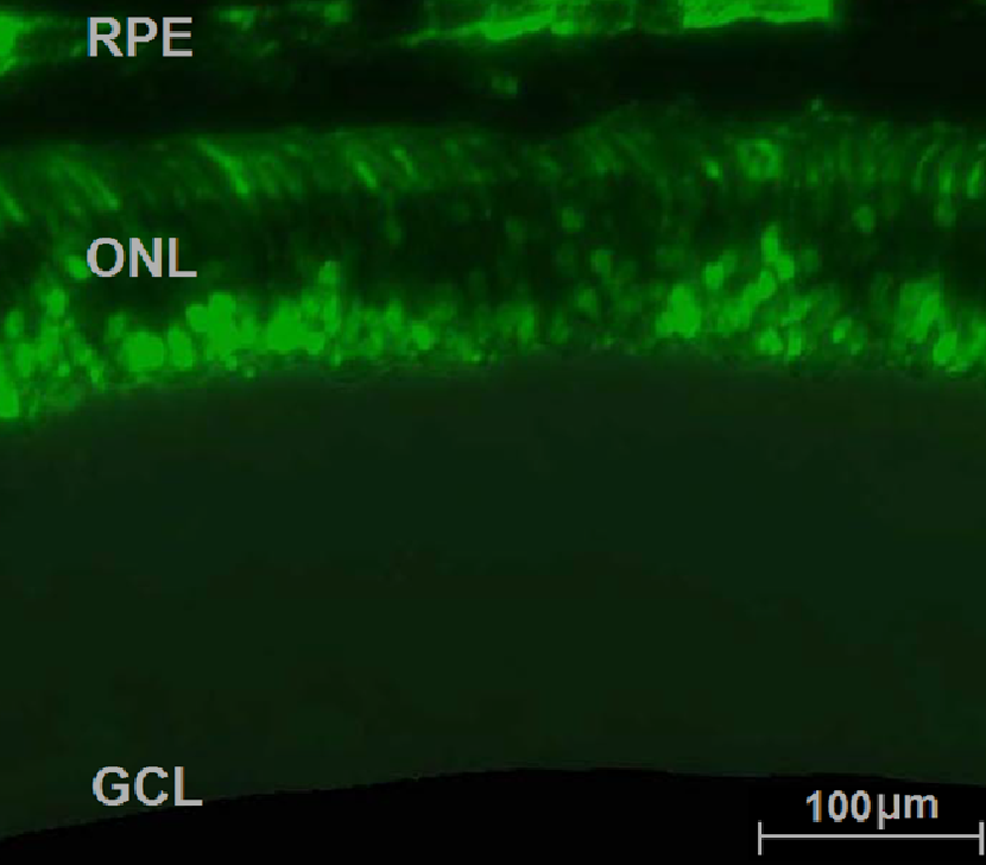

Supplement: S1 Fig — (TIF) [file pone.0156542.s001.tif]
